# An Experimental Performance Assessment of Temporal Convolutional Networks for Microphone Virtualization in a Car Cabin

**DOI:** 10.3390/s24165163

**Published:** 2024-08-10

**Authors:** Alessandro Opinto, Marco Martalò, Riccardo Straccia, Riccardo Raheli

**Affiliations:** 1 Keysight Technologies Italy S.r.l., 20127 Milan, Italy; 2 Department of Electrical and Electronic Engineering, University of Cagliari, 09124 Cagliari, Italy; marco.martalo@unica.it; 3 National Inter-University Consortium for Telecommunications (CNIT), 09123 Cagliari, Italy; 4 ASK Industries S.p.A., 42124 Reggio Emilia, Italy; 5 Department of Engineering and Architecture, University of Parma, 43124 Parma, Italy

**Keywords:** virtual microphone technique, temporal convolutional network, neural networks, active noise control, automotive

## Abstract

In this paper, the experimental results on microphone virtualization in realistic automotive scenarios are presented. A Temporal Convolutional Network (TCN) was designed in order to estimate the acoustic signal at the driver’s ear positions based on the knowledge of monitoring microphone signals at different positions—a technique known as virtual microphone. An experimental setup was implemented on a popular B-segment car to acquire the acoustic field within the cabin while running on smooth asphalt at variable speeds. In order to test the potentiality of the TCN, microphone signals were recorded in two different scenarios, either with or without the front passenger. Our experimental results show that, when training is performed in both scenarios, the adopted TCN is able to robustly adapt to different conditions and guarantee a good average performance. Furthermore, an investigation on the parameters of the Neural Network (NN) that guarantee the sufficient accuracy of the estimation of the virtual microphone signals while maintaining a low computational complexity is presented.

## 1. Introduction

The control of the acoustic field in a car cabin is gaining momentum in the research community due to its industrial relevance to provide new applications, ranging from the comfort of the driver and passengers to innovative entertainment applications. These methods are based on the installation of microphones to capture the sound inside the cabin. However, the placement of these microphones is highly constrained by the car structure and production costs. Therefore, studying the reconstruction of the audio from spatially placed microphones, like in microphone virtualization, is of paramount importance.

Microphone virtualization, also known in the literature as the Virtual Microphone Technique (VMT) or Remote Microphone Technique (RMT), as part of a general family of virtual sensing approaches [1], refers to the technique of reconstructing an acoustic signal by employing monitoring microphones placed at some distance from the acoustic zone to be controlled. A possible solution in VMT applications is obtained by modeling virtual microphone acoustic characteristics like directivity or sensitivity [2,3]. The VMT is of extreme interest in several audio-based applications. In [4], the VMT is applied to Perceptual Soundfield Reconstruction (PSR) methods to improve their accuracy in a reproduction system employing a small number of audio channels. Another application area of the VMT is in conjunction with binaural noise reduction systems to simultaneously increase the noise reduction performance and the preservation of the binaural noise cues, as conducted in [5].

One of the key applications where the VMT may be effective is Active Noise Control (ANC). In [6], the VMT is employed in a multichannel ANC headrest aimed at creating quiet areas at the passenger positions within the cabin of a public transport vehicle. The key aspect of this work is the use of a distributed implementation to avoid the high computational demands of the multichannel system. The use of the VMT to increase the robustness of the active headrests is exploited in [7,8]. The problem of the optimal controller design with virtual sensing, e.g., in ANC systems, is investigated in [9].

As mentioned, the idea behind the VMT is to reconstruct the signals regarding the so-called virtual microphones by exploiting the monitoring ones. In fact, microphone virtualization is usually based on the use of an observation filter (OF) [10,11,12], i.e., the physical acoustic channel between monitoring and virtual microphones. In the literature, various OF estimation approaches were proposed and employed [13]. Among them, the most relevant OF estimation algorithms are those in [14,15,16]. Furthermore, “additional” or “auxiliary” filter-based methods were developed in [17,18,19] as alternatives to OFs.

OFs are used to process the audio waves acquired by the monitoring microphones in order to estimate the acoustic field at the virtual positions. In principle, an ANC system enables minimizing the disturbing audio at the virtual locations. A preliminary phase exists in which physical microphones are positioned at the desired quiet zones, which is necessary in order to acquire the virtual microphone signals and measure the physical channels from the monitoring to virtual microphones. Note that the term “virtual microphone” is used throughout the manuscript to interchangeably denote either the true virtual signal to be reconstructed during the operation or the signal acquired by a temporarily positioned physical microphone during the system training. The context should eliminate any ambiguity. Hence, the necessary observation filters are estimated off-line, and therefore they are only able to model time-invariant channels. For this reason, the system may be sub-optimal if the actual channel is time-varying, as in the automotive scenario, where the acoustic channel may change, e.g., depending on the user’s head movement, weather, or driving conditions. The estimation accuracy of the virtual microphone signals is limited by a distance–bandwidth trade-off: the higher the frequency, the harder the task if the monitoring microphones are too far away from the virtual positions. In fact, in order to obtain the best possible accuracy, the monitoring microphones have to be installed as close as possible to the target quiet zones.

In recent years, the success and popularity of machine learning frameworks have increased [20]. In particular, due to the growing interest in deep learning techniques in several domains, there has been considerable interest in machine learning frameworks in the audio signal processing community [21,22]. The main advantage of Deep Neural Networks (DNNs) is that they can be trained to perform complex operations through supervised learning [23]. As in several other fields, Neural Networks (NNs) have proven to be successful in the audio domain for several tasks, such as acoustic modeling, source separation, sound event recognition, speech recognition, music classification, and generative audio [22,24]. Motivated by the success of these works, a DNN, based on a Temporal Convolutional Network (TCN) architecture [25], is proposed in this work for the microphone virtualization task. In particular, we focus on automotive applications, in which the users cannot wear headsets for reasons of safety and comfort. Therefore, the monitoring microphones can only be placed in the vicinity of the zones to be silenced (the virtual positions), e.g., the driver’s/passenger’s ears.

In order to acquire microphone signals, an experimental measurement campaign was performed on a popular B-segment car running on a closed path with smooth asphalt and variable speeds. In particular, six monitoring microphones were installed at the roof and at the driver’s sun visor of the car, and two other microphones were positioned around the left and right driver’s ears for the purpose of virtual signal acquisition to be used for training. In fact, we aim at reconstructing the acoustic field at the driver’s ears by using microphones placed at various positions inside the car cabin. Since the TCN may be used in the realm of classification problems, two different acquisition scenarios were considered, either with or without the front passenger. In fact, the presence or absence of the passenger is expected to modify the responses of the various acoustic channels inside the car cabin.

Our experimental results show that training the TCN on both scenarios (with and without the passenger) leads to robust system performance in terms of signal reconstruction under different conditions. Moreover, a comprehensive performance investigation under different parameters of the NN is presented. The trade-off between the signal reconstruction accuracy and the system computational complexity is also discussed.

The rest of this paper is organized as follows. The general system model is presented in Section 2. In Section 3, the employed TCN architecture is presented. The description of the experimental measurement campaign and scenarios is provided in Section 4. The experimental numerical results are presented and discussed in Section 5. Finally, in Section 6, the concluding remarks are drawn.

## 2. System Model

**Figure 1:**
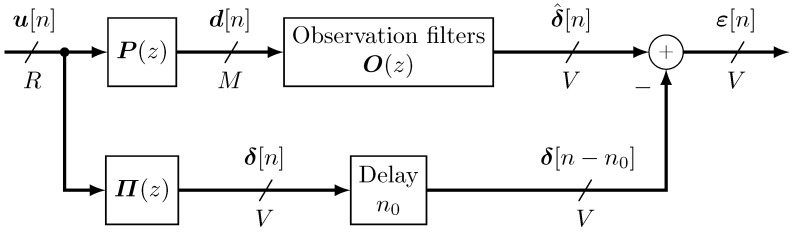
Block diagram of car interior sound propagation detected by virtual and monitoring microphones and reconstructed by observation filters.

The VMT can be approached as a filter identification problem as depicted in Figure 1. In fact, the estimation of the observation filters, which represent the acoustic paths between monitoring and virtual microphones, is needed in order to reconstruct the virtual microphone signals starting from the monitoring ones.

The propagation of air-borne and structure-borne sound waves in the car interior is detected by the monitoring and virtual microphones. This can be represented as a discrete convolution between an unknown *R*-length vector signal $u\left[n\right]={\left[u_{1}\left[n\right],u_{2}\left[n\right],\ldots ,u_{R}\left[n\right]\right]}^{T}$, ${\left(\cdot \right)}^{T}$ being the transpose operator, and the so-called primary paths represented by the blocks P(z) and Π(z). For notational clarity, Latin letters denote the physical primary path from the (acoustic) noise sources to the monitoring microphones, as well as the signals acquired at them. Greek letters, instead, denote the virtual primary path from the (acoustic) noise sources to the virtual microphones, as well as the signal acquired at them. The primary paths can be modeled as Finite Impulse Response (FIR) filters and encompass the information on the physical transformation that the cabin environment applies to the disturbing signals u[n] before they are acquired by the microphones. Thus, the *i*-th monitoring and *j*-th virtual microphone signals can be, respectively, expressed as

(1) $$\begin{array}{cc} d_{i}\left[n\right] & =\sum_{\ell =1}^{R}u_{\ell }\left[n\right]⊗p_{i\ell }\left[n\right]\;i=1,2,\ldots ,M \end{array}$$

(2) $$\begin{array}{cc} \delta_{j}\left[n\right] & =\sum_{\ell =1}^{R}u_{\ell }\left[n\right]⊗\pi_{j\ell }\left[n\right]\;j=1,2,\ldots ,V \end{array}$$

where ⊗ denotes the convolution and $p_{i\ell }\left[n\right]$ and $\pi_{j\ell }\left[n\right]$ represent the impulse responses from the *ℓ*-th audio source to the *i*-th monitoring and *j*-th virtual microphone signals, respectively, and are associated with the transfer functions P(z) and Π(z), respectively.

In order to take into account the possible propagation delay between the monitoring and virtual microphone signals, as suggested in [16,26,27], a delay is introduced in the virtual microphone signals so that causal observation filters may be effective. As depicted in Figure 1, the error signals, defined as the difference between virtual signals and their retrieved version, can thus be compactly expressed as

(3) $$\varepsilon \left[n\right]=\hat{\delta }\left[n\right]-\delta \left[n-n_{0}\right]$$

where $n_{0}$ denotes the introduced delay. Obviously, the better the estimation, the smaller the error signal.

## 3. TCN Model

### 3.1. TCN Architecture

TCNs [25] represent a powerful Neural Network architecture to approach the problems of classification, estimation, and generation of data in the temporal domain. They are an alternative to Recurrent Neural Networks (RNNs), typically used to process sequences (such as audio signals [28]), and use a Convolutional Neural Network (CNN) architecture, which features the advantage of handling parallelizable computation, resulting in faster training speed [29]. TCNs have proved to be successful also when adopted for speech enhancement [30,31,32], sound localization [33], speech synthesis, and raw audio generation [24]. Unlike the majority of other CNN models, typically built for image data, which require some sort of pre-processing, such as the Short-Time Fourier Transform, to obtain 2-D spatial data, TCNs for audio data do not require any pre-processing of the input since the network performs convolutions directly on the time representation of the audio data. The terminology used in the remainder of this paper to describe the TCN architecture follows that in [24], to which the reader is referred.

The layers that compose a TCN are called *residual blocks*, which perform several parallel *dilated* convolutions followed by non-linear *activations* in order to perform some filtering of the input (or extract some features in the case of classification). The output of each layer becomes the input of the following one, but the intermediate result is also extracted in order to further process the features or perform the filtering at different temporal scales.

The dilated convolution operation between two time sequences x[n] and h[n] can be denoted by the symbol ${⊗}^{\tau }$ and is defined as

(4) $$y\left[n\right]=x\left[n\right]{⊗}^{\tau }h\left[n\right]≜\sum_{m=-\infty }^{\infty }x\left[m\right]h\left[n-\tau m\right]$$

where τ is a stride factor on the sequence, referred to as *dilation* and increasing exponentially with the depth *ℓ* of the network in which the residual block is operating, i.e., $\tau =2^{\ell -1}$, ℓ=1,2,…,L, *L* being the maximum layer depth. The presence of the stride allows the network to increase its receptive field, which represents the length of the input sequence window that contributes to produce a single output sample without increasing the number of samples used in the convolution. As a side effect, it also provides, as an intermediate output of each layer, the input data processed at different time scales. The maximum receptive field size (at maximum layer depth *L*, i.e., the total number of residual blocks) can be calculated as [34]

(5) $$r_{f}=\left(\tau -1\right)\left(\tau^{L}-1\right)+1.$$

**Figure 2:**
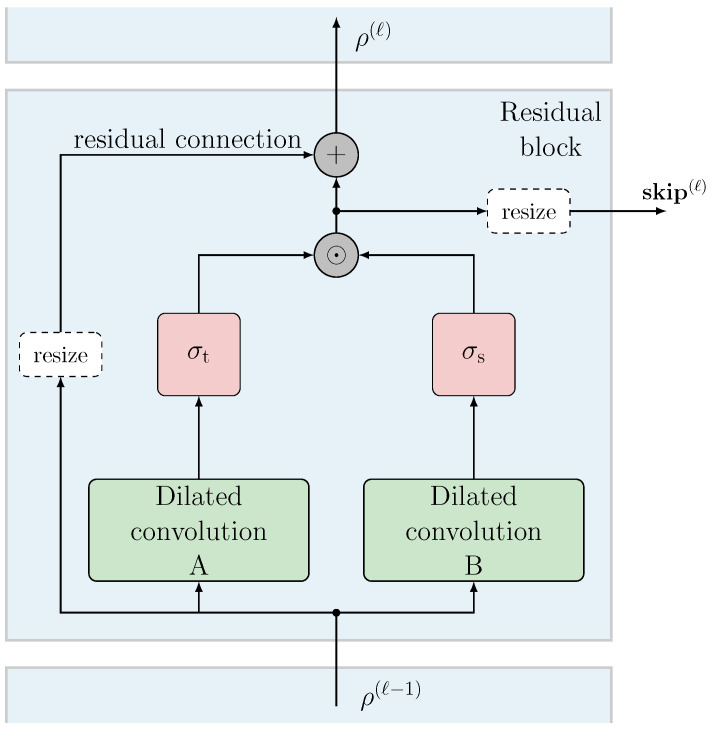
Representative scheme of the residual block employed in the considered model.

The schematic in Figure 2 represents a residual block at the *ℓ*-th layer. The input of the residual block is the output of the previous block (at the (ℓ−1)-st layer). The first residual block of the network, which has index ℓ=1, instead uses directly the samples of the given input sequence. The input of the layer, denoted as $\varrho^{\left(\ell -1\right)}$, is processed by the so-called neurons, which are activated through connections weighted by a vector of parameters μ, usually called weights.

The residual block is composed of two dilated convolutions with dilation $\tau =2^{\left(\ell -1\right)}$, where each one performs several parallel convolutional filtering operations. Each convolution filter aims at learning a different set of weights through a backpropagation algorithm. The number of convolutions executed in parallel in a residual block is referred to as the *feature size* of that block. After each convolution, a non-linear layer is applied; in particular, two different functions are used for each dilated convolution: the hyperbolic tangent activation $\sigma_{t}\left(x\right)$ and a sigmoid activation $\sigma_{s}\left(x\right)$, respectively, defined as

$$\begin{array}{ccc} \sigma_{t}\left(x\right) & = & \frac{e^{x}-e^{-x}}{e^{x}+e^{-x}}\\ \sigma_{s}\left(x\right) & = & \frac{1}{e^{-x}+1}. \end{array}$$

For each pair of convolutions, after the activation, the term-by-term product is taken. Then, a so-called *skip connection* is employed to use the result of this operation, eventually resized if needed, as the output of the residual block. Moreover, the input data of the block are summed again at the output through the residual connection. This generates the data used at the input of the following residual block, denoted by $\rho^{\left(\ell \right)}$. Optionally, before this sum, a resize operation may be required to match feature sizes between two adjacent residual blocks.

**Figure 3:**
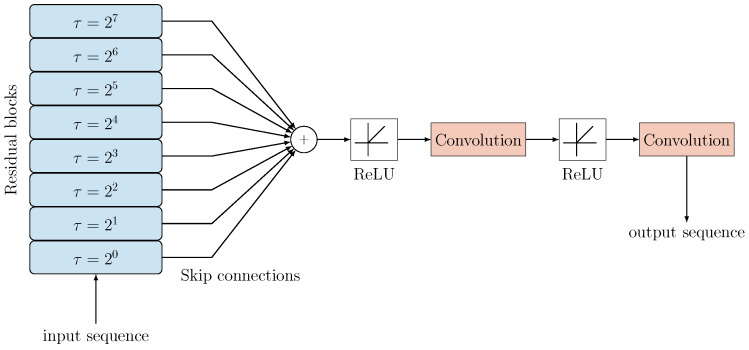
Example scheme representing the considered TCN model. The layered residual blocks (with L=8) on the left generate individual outputs, which are summed and rescaled to obtain the output sequence.

The overall structure of the Neural Network is shown in Figure 3. All the skip connections, which carry the data processed using different dilation coefficients, therefore applying non-linear filters at different time scales, converge to a sum node. The output of this sum is then processed by a section consisting of Rectified Linear Units (ReLUs) activation functions and final convolutions. These have the role of compressing the meaningful information extracted by the residual blocks by reducing the size of the input matrix back to a single vector, which represents the output of the network.

### 3.2. TCN Implementation

The relevant hyperparameters for the TCN implementation, related to its size structure, are the following:

- the number of TCN layers *L*, i.e., the number of stacked residual blocks;
- the feature size of each residual block, i.e., the number of filters that are calculated in parallel at each layer;
- the filter sizes in the convolution operations at the end of the network;
- the total number of training epochs.

The backpropagation learning algorithm for the vector of TCN parameters, referred to as μ, follows a gradient descent update rule of type

$$\mu \left[e+1\right]=\mu \left[e\right]-\tau_{\mu }\nabla_{\mu }\;\varepsilon \left[e\right]$$

where *e* denotes the epoch index, $\nabla_{\mu }$ is the gradient operator with respect to the parameter vector, and $\tau_{\mu }\in \left(0,1\right)$ is a learning rate drop factor. The recursion is initialized at epoch e=1 with $\mu \left[1\right]=\mu_{1}$; i.e., the vector has all elements equal to $\mu_{1}$.

The selection of the parameters is performed based on the estimated minimum size of the required receptive field (which affects the required number of layers and the size of the convolutions). Then, preliminary learning tests are performed and, finally, the computing resources available for the learning process, which typically act as bottlenecks on the model size, are heuristically determined.

## 4. Experimental Setup and Scenarios

**Figure 4:**
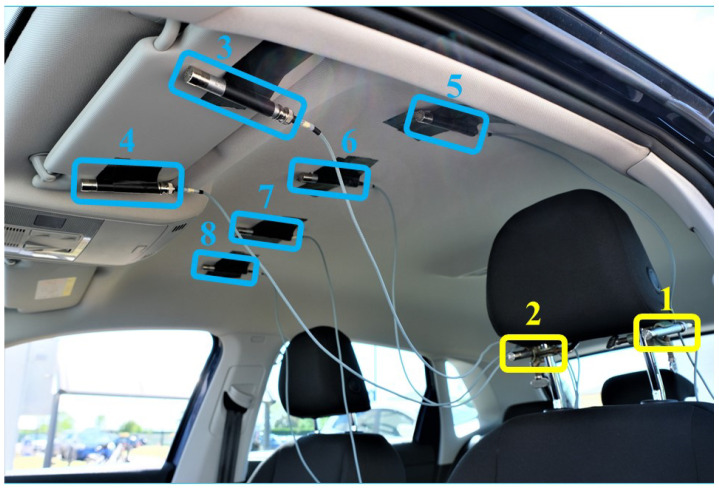
Microphone installation within the car cabin interior: virtual, at the driver headrest (in yellow, labels 1–2), and monitoring microphones, at the driver sun visor and roof (in blue, labels 3–8).

The employed microphone signals are obtained by an experimental measurement campaign performed on a popular B-segment car. Microphone signals were acquired by using a well-known professional portable multi-track field recorder with 8 channels, namely the ZOOM F8 [35], with a sample rate of 48 kHz. In order to acquire the monitoring microphone signals, six Brüel & Kjær transducers for measurements in transport-noise with a sensitivity of 31.6 mV/Pa were installed within the car cabin at the roof and at the driver’s sun visor. Figure 4 shows a photo of the installation of virtual (in yellow) and monitoring (in blue) microphones within the car cabin; note that this setup is similar to that presented in [27]. More precisely, microphones 3 and 4 were placed at the left and right edges of the driver’s sun visor, respectively, whereas, at the cabin roof, from left to right, microphones 5, 6, 7, and 8 were positioned. Note that the distance between contiguous microphones is about 25 cm. Similarly, for the acquisition of the virtual microphone signals, two other Brüel & Kjær microphones were placed around the driver’s left and right ears, i.e., just below the headrest at the maximum possible height.

The rationale of the microphones’ positions is the following. Virtual microphones correspond to the driver’s ears since the goal is to reconstruct the audio content at those positions. On the other hand, the positions of the monitoring microphones are determined as a reasonable trade-off between system performance and complexity. In fact, the larger the number of sufficiently spaced microphones, the better the sound reconstruction. Moreover, the chosen positions (i.e., car roof and driver’s sun visor) are such that the setup has some practical relevance since a final prototype can be easily installed.

**Figure 5:**
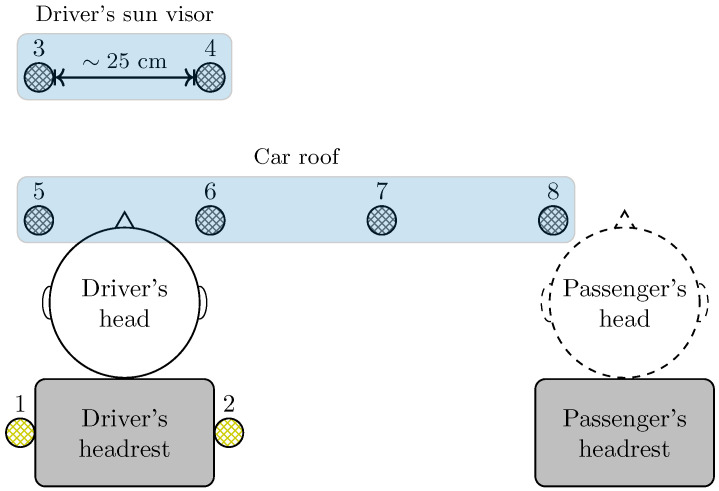
Representative scheme of experimental microphone installation within car cabin and scenario.

The environmental acoustic waves propagating inside the car were recorded for about 5 min while the car was running on a closed path on smooth asphalt at variable speeds, according to traffic conditions, from 40 km/h to 90 km/h. In order to test the potential of the employed TCN, based on the presence or absence of the front passenger, two different scenarios were considered, namely driver alone (scenario A) and presence of the front passenger (scenario P). This yields a pair of two microphone recordings in which, within the car, only the driver is present in scenario A and both the driver and passenger are present in scenario P. The idea is to avoid the overfitting phenomenon [36] of the TCN by feeding the network with signals having a reasonable diversity among them since the road trip, driving scenario, and setup remained unchanged between scenarios A and P. In fact, the primary paths change if the car cabin environment undergoes alterations, i.e., the absence or presence of the passenger. If the primary paths vary, microphone signals change. A representative scheme of considered setup and scenarios is shown in Figure 5, in which the front passenger is depicted by dashed lines according to his presence/absence. To make our analysis as realistic as possible, we allow both the driver and the passenger to freely move during the acquisition inside the car. Therefore, the obtained results can be considered as representative of a realistic scenario with different occupants’ characteristics.

This set of microphone acquisitions is used for the training and testing tasks of the TCN, which aim at the virtualization of the driver’s ear signals based on the knowledge of the monitoring microphone signals. We consider five possible scenarios, as summarized in Table 1. First, we use the same scenario (namely A or P) for both training and testing. These scenarios are referred to as “direct” in Table 1. Then, with the aim of analyzing the robustness of the network, we train the TCN in one scenario (A or P, respectively) and test it in the other one (P or A, respectively), thus obtaining the so-called mismatched scenarios, referred to as “cross” in Table 1. Finally, by shuffling the sets of these two acquisitions (A and P) in a single recording, one receives a “mixed” case, namely the scenario M.

**Figure 6:**
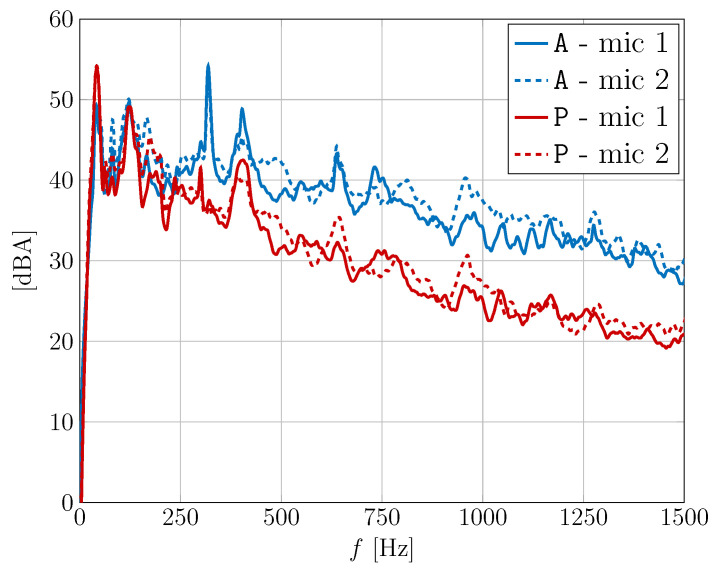
A-weighted spectra of the signals at microphones 1 and 2 for the considered scenarios without and with passenger.

In order to preliminarily investigate the audio characteristics in the considered settings, in Figure 6, the A-weighted spectra of the signals at microphones 1 and 2 are shown for the considered scenarios without and with passenger.

This figure can illustrate the “average” differences among the spectra under different conditions, where average means with variable car speeds in the considered range 40–90 km/h. It can be seen that, apart from possibly different audio levels due to different calibration settings, the spectra in the two cases show some differences, for a fixed microphone, especially above 250 Hz range, due to different acoustic paths incurred by the presence or absence of the passenger. Specifically, some peaks possibly caused by strong reflections, e.g., about 300 Hz, are significantly attenuated by the presence of the passenger, which, in general, seems to reduce the amount of reverberation. Therefore, it is expected that the proposed TCN-based architecture may exploit its robustness in these conditions.

We may expect that microphone virtualization is less effective when a mismatched cross-scenario is considered, e.g., A vs. P, with respect to the matched case, e.g., A vs. A. However, system performance may increase if case M is taken into consideration. In fact, since during the training period the TCN is exposed to both scenarios, the network may be robust against different operational conditions, yielding good performance, on average, in both cases.

Finally, note that proper training is crucial to allow the TCN to be effective in the considered scenario. In particular, training should be repeated for each considered car cabin. To this end, a sufficiently large number of audio samples, representative of the considered vehicle, need to be collected in this phase to finetune the algorithm.

## 5. Numerical Results

In this section, some results on microphone virtualization performed by using the TCN described in Section 3 are presented and discussed. For the sake of computational complexity saving, microphone signals are down-sampled by a factor 16, thus yielding a sample rate $f_{s}=3$ kHz. Numerical results are assessed in terms of Mean Square Error (MSE) normalized with respect to the mean square value of the target signal. More precisely, for the *v*-th virtual microphone, the normalized MSE $\Upsilon_{v}$, in dB, is defined as

(6) $$\Upsilon_{v}=10\log_{10}\frac{\sum_{n=0}^{N-1}{\left(\delta_{v}\left[n-n_{0}\right]-{\hat{\delta }}_{v}\left[n\right]\right)}^{2}}{\sum_{n=0}^{N-1}\delta_{v}^{2}\left[n-n_{0}\right]}\;\;\;\left[\mathrm{dB}\right]$$

where *N* is the considered time window length, $\delta_{v}\left[n\right]$ is the *v*-th virtual microphone signal, and ${\hat{\delta }}_{v}\left[n\right]$ is its reconstructed version by means of the TCN. The smaller the value of $\Upsilon_{v}$, the better the performance. Note that, in (6), the target signal is delayed regarding $n_{0}$ samples as discussed in Section 2. The optimal delay $n_{0}$ can be empirically found by using a brute-force search or can be approximated by estimating the cross-correlation between the monitoring and virtual signals.

The 5 min recordings illustrated in Section 4 are divided so that 80% are used for training and 20% for testing. This means that 4 min of each recording are used for TCN training and 1 min for performance validation.

In the remainder of this section, only the right virtual position (microphone number 2) is analyzed as similar considerations hold for the left one. As a preliminary analysis, we investigate the problem of setting the hyperparameters in TCN. This analysis often requires an extensive brute-force approach to find the best-performing set of values, which, from a simulation time viewpoint, involves a non-negligible cost. For this reason, a set of hyperparameters was chosen after a reasonable number of attempts. A comparison on the number of non-linear layers (*L*) of the TCN to be employed was also pursued as the number of employed non-linear layers in the TCN may impact the system performance. Four values of non-linear layers *L* were considered: 2, 5, 7, and 10. The other used parameters are summarized in Table 2.

**Figure 7:**
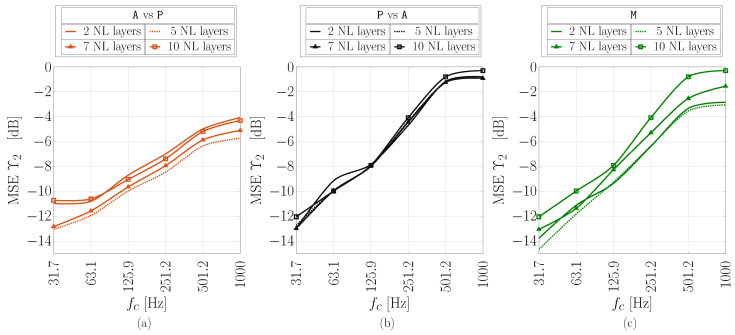
MSE analysis as a function of 1-octave bands against the number of non-linear blocks of the considered TCN for different scenarios: (**a**) A vs. P, (**b**) P vs. A, and (**c**) M.

The estimation accuracy performance in terms of normalized MSE as a function of 1-octave bands for the considered four values of non-linear layers *L* is shown in Figure 7 for different scenarios: (a) A vs. P, (b) P vs. A, and (c) M. For the octave band analysis, signals in  (6) are decomposed into 1-octave sub-bands [37]. In particular, second-order octave filters were used in this analysis. Note that only the cross and mixed cases are depicted here since they are more significant with respect to the direct ones, i.e., A vs. A and P vs. P, in applications.

It is possible to observe that, as expected, estimation accuracy decreases as the frequency increases, regardless of the scenario taken into consideration. Except for the P vs. A scenario, for which system performance seems not affected by the employed number of non-linear layers *L*, as the curves almost overlap with each other, it may be noticed that a good trade-off between accuracy and computational complexity is L=5. Moreover, interesting results are obtained for the M scenario. In fact, since the TCN is trained by using recordings associated with both cases, it shows robustness to the scenario, thus obtaining fairly good accuracy performance. We might interpret the network behavior as if it is able to intrinsically classify the scenario.

**Figure 8:**
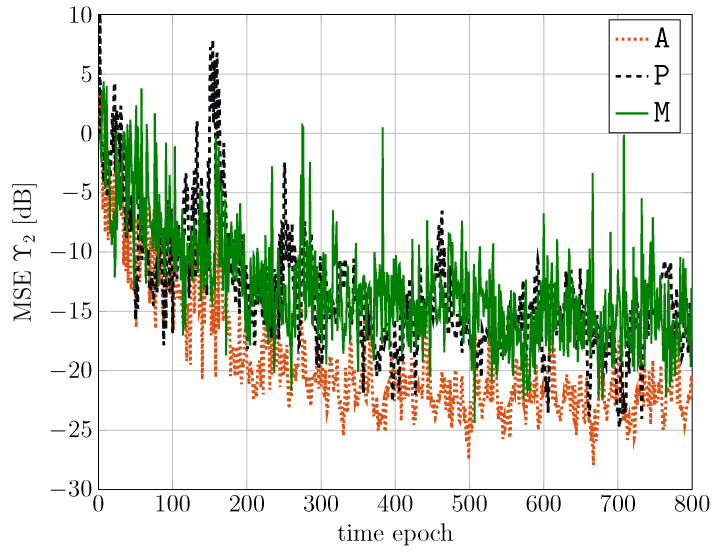
Normalized MSE during the training phase, as a function of the time epoch, for the considered scenarios.

For L=5 non-linear layers, a deeper MSE analysis is now presented for all the scenarios considered in Table 1. In Figure 8, the normalized MSE during the training phase is shown, as a function of the time epoch, for the considered scenarios.

It can be observed that, in all cases, after 300 epochs, the MSE is almost at convergence, even if some fluctuations can still appear due to the time-varying characteristics of the signals. Moreover, the scenario without passenger (i.e., A, with dotted line) achieves a lower MSE in the training phase with respect to that with the passenger (i.e., P, with dashed line). Finally, the mixed case is limited by the worst case, i.e., the passenger scenario.

**Figure 9:**
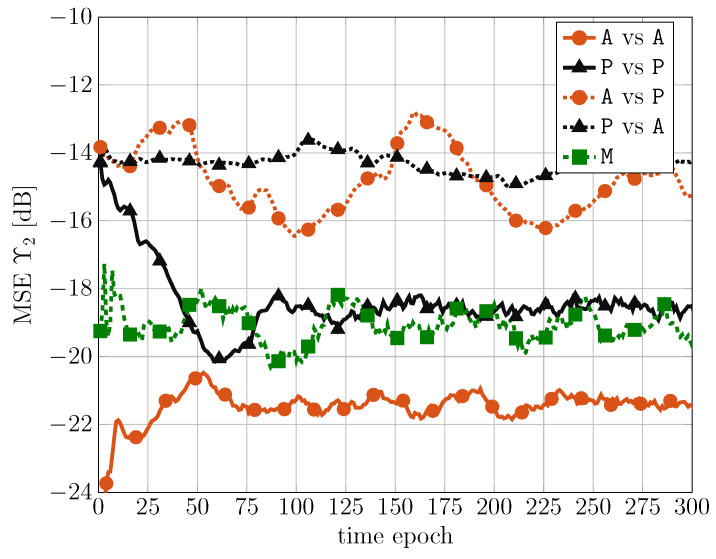
Normalized MSE during the testing phase, as a function of the time epoch, for the considered scenarios.

In Figure 9, the normalized MSE during the testing phase is shown, as a function of the time epoch, for the considered scenarios. Note that in all cases a standard moving average over 30 time epochs is performed to reduce random fluctuations.

It is worth noting that in this case the normalized MSE is smaller than that in Figure 8 during the training phase. Moreover, as expected, the matched cases (i.e., A vs. A and P vs. P) achieve a normalized MSE smaller than that of the mismatched cases (i.e., A vs. P and P vs. A). However, it is interesting to observe that the mixed case (i.e., M) has intermediate performance (closer to the worst matched scenario). Since the mixed scenario is characterized by a composition of alone and passenger scenarios, this means that the considered TCN has intrinsic robustness against various scenario conditions.

**Figure 10:**
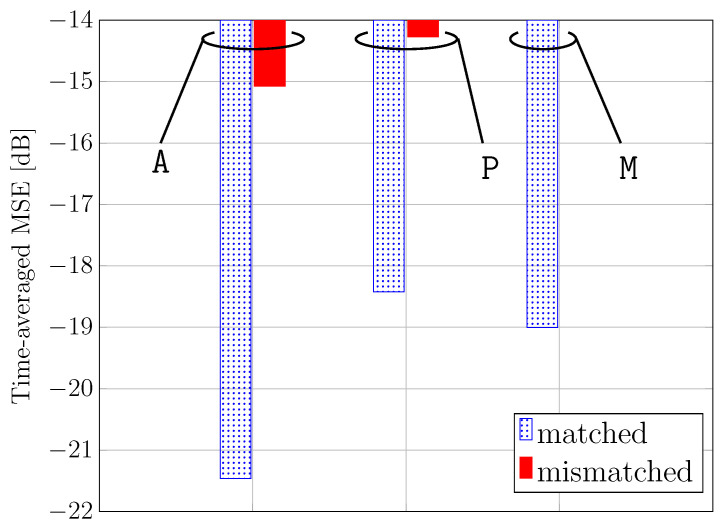
Time-averaged normalized MSE during the testing phase for all the considered scenarios.

Finally, in Figure 10, the time-averaged normalized MSE during the testing phase is shown for all the considered scenarios.

The considerations carried out regarding Figure 9 are confirmed in Figure 10. It is worth noting that the mixed case can achieve satisfactory accuracy on average in all cases since the considered TCN architecture is robust against various scenario conditions due to its inherent classification capabilities. In particular, a sufficiently small MSE of −19 dB is obtained.

## 6. Concluding Remarks

In this paper, a TCN-based solution for VMT applications is investigated. A virtualization system model was developed and simulations were performed on realistic microphone signals in an automotive environment. In particular, an experimental measurement campaign was conducted on a B-segment car in order to acquire six monitoring and two virtual microphone signals for a few driving scenarios. With the aim of testing the robustness of the TCN, five different scenarios, depending on the presence/absence of the front passenger and different road conditions, were considered. Various setups of the TCN were analyzed and a number of non-linear layers that maximize the performance were found. Even if the size of the collected data may be a limiting factor, one can conclude that the proposed method achieves significant performance for all the considered scenarios. Moreover, our results show the inherent TCN robustness in the mixed scenario. Therefore, our approach appears to be promising since it may enable “universal” signal estimation in the VMT setting with satisfactory accuracy in different (mismatched) scenarios. This may lead to significant savings in the complexity of the designed signal processing system in realistic automotive scenarios. The potential practical application of this approach is in providing effective microphone virtualization in time-varying scenarios, like the discussed absence/presence of a passenger, provided a representative subset of scenarios are considered during training. The experimental investigation of this aspect is beyond the scope of the present paper and is left as future work. 

## Figures and Tables

**Table 1:** Possible combinations of scenarios for the training and testing of the TCN.

Name	Training	Validation	Type
A vs. A	alone	alone	direct
A vs. P	alone	passenger	cross
P vs. A	passenger	alone	cross
P vs. P	passenger	passenger	direct
M	alone and passenger	alone and passenger	mixed

**Table 2:** Summary of the used hyperparameters.

Maximum number of TCN layers *L*	10
Feature size at each layer	100
Training epochs	5
Starting weight recursion $\mu_{1}$	0.005
Drop factor $\tau_{\mu }$	0.25
Filter size *D* for residual block convolutions	2
Post-sum convolution feature size	256

## Data Availability

Data are contained within the article.
